# Oligonucleotides Targeting DNA Repeats Downregulate *Huntingtin* Gene Expression in Huntington's Patient-Derived Neural Model System

**DOI:** 10.1089/nat.2021.0021

**Published:** 2021-12-10

**Authors:** Tea Umek, Thomas Olsson, Olof Gissberg, Osama Saher, Eman M. Zaghloul, Karin E. Lundin, Jesper Wengel, Eric Hanse, Henrik Zetterberg, Dzeneta Vizlin-Hodzic, C. I. Edvard Smith, Rula Zain

**Affiliations:** ^1^ Department of Laboratory Medicine, Clinical Research Center, Karolinska Institutet, Karolinska University Hospital Huddinge, Huddinge, Sweden.; ^2^ Department of Physiology, Institute of Neuroscience and Physiology, the Sahlgrenska Academy at the University of Gothenburg, Gothenburg, Sweden.; ^3^ Department of Clinical Pathology and Cytology, Sahlgrenska University Hospital, Gothenburg, Sweden.; ^4^ Department of Pharmaceutics and Industrial Pharmacy, Faculty of Pharmacy, Cairo University, Cairo, Egypt.; ^5^ Department of Pharmaceutics, Faculty of Pharmacy, Alexandria University, Alexandria, Egypt.; ^6^ Department of Physics, Chemistry and Pharmacy, Biomolecular Nanoscale Engineering Center, University of Southern Denmark, Odense M, Denmark.; ^7^ Department of Psychiatry and Neurochemistry, Institute of Neuroscience and Physiology, the Sahlgrenska Academy at the University of Gothenburg, Gothenburg, Sweden.; ^8^ Department of Neurodegenerative Disease, Institute of Neurology, University College London, London, United Kingdom.; ^9^ Clinical Neurochemistry Laboratory, Sahlgrenska University Hospital, Mölndal, Sweden.; ^10^ UK Dementia Research Institute at UCL, London, United Kingdom.; ^11^ Department of Clinical Genetics, Center for Rare Diseases, Karolinska University Hospital, Stockholm, Sweden.

**Keywords:** gene therapy, anti-gene, antisense, human induced pluripotent stem cells, gymnosis

## Abstract

Huntington's disease (HD) is one of the most common, dominantly inherited neurodegenerative disorders. It affects the striatum, cerebral cortex, and other subcortical structures leading to involuntary movement abnormalities, emotional disturbances, and cognitive impairments. HD is caused by a CAG•CTG trinucleotide-repeat expansion in exon 1 of the *huntingtin* (*HTT*) gene leading to the formation of mutant HTT (mtHTT) protein aggregates. Besides the toxicity of the mutated protein, there is also evidence that mt*HTT* transcripts contribute to the disease. Thus, the reduction of both mutated mRNA and protein would be most beneficial as a treatment. Previously, we designed a novel anti-gene oligonucleotide (AGO)-based strategy directly targeting the *HTT* trinucleotide-repeats in DNA and reported downregulation of mRNA and protein in HD patient fibroblasts. In this study, we differentiate HD patient-derived induced pluripotent stem cells to investigate the efficacy of the AGO, a DNA/Locked Nucleic Acid mixmer with phosphorothioate backbone, to modulate *HTT* transcription during neural *in vitro* development. For the first time, we demonstrate downregulation of *HTT* mRNA following both naked and magnetofected delivery into neural stem cells (NSCs) and show that neither emergence of neural rosette structures nor self-renewal of NSCs is compromised. Furthermore, the inhibition potency of both *HTT* mRNA and protein without off-target effects is confirmed in neurons. These results further validate an anti-gene approach for the treatment of HD.

## Introduction

Huntington's disease (HD) is one of the most common dominantly inherited neurodegenerative disorders. It affects the striatum, cerebral cortex, and other subcortical structures, leading to clinical symptoms such as involuntary movement abnormalities, emotional disturbance, and cognitive impairment. The therapies currently available to HD patients offer only moderate symptom relief, and the affected individuals typically die 15–20 years postdiagnosis due to complications such as pneumonia, dysphagia, heart disease, or suicide.

HD is caused by a dominant mutation, an expansion of CAG•CTG trinucleotide-repeat in exon 1 of the *huntingtin* gene (*HTT*), leading to the formation of mutant HTT (mtHTT) protein that aggregates in the nucleus and cytoplasm of striatal and cortical neurons, disrupting important cellular functions [1,2]. The toxic gain of function of mtHTT is generally considered as the primary cause of disease [3,4], and accordingly, it has been shown in mice that deleting the expanded allele or decreasing the expression of the protein can halt the progression of HD [5,6]. Furthermore, several studies suggest that reducing mtHTT, as well as wild-type HTT (wtHTT), is well tolerated in adult mice and larger animals [4,7–9], but the loss of wtHTT is lethal to the mouse embryo [10]. However, in a human embryonic stem cell-derived neuronal model, decreasing wtHTT by 90% does not affect normal phenotype, whereas a 10%–20% reduction of the mtHTT alone is sufficient to result in a significant reduction of toxicity [11]. Thus, lowering mtHTT at the expense of a partial loss of wtHTT seems to be acceptable. This has led to the development of several promising disease-modifying oligonucleotides (ONs), which entered clinical trials, aiming for the degradation of the mRNA [12]. Even if Tominersen (formerly known as IONIS-HTT_RX_), a non-allele-specific antisense ON (ASO), did not exhibit any beneficial clinical effects, it resulted in a dose-dependent reduction of mtHTT in participants' cerebrospinal fluid [13].

Besides the toxicity of the mutated protein, an increasing body of evidence indicates that mt*HTT* mRNA contributes to striatal and cortical atrophy [14]. RNA stable hairpin structures, formed in the CAG•CTG expanded region, interact with specific proteins leading to dysregulated splicing [3,15–18]. Furthermore, secondary structures formed by the repeats can be cleaved by Dicer producing toxic small RNAs [15,19]. Thus, targeting the most proximal cause of the disease, the *HTT* gene itself, thereby affecting both of the abovementioned disease-causing macromolecules, may be the most beneficial form of treatment. Previously, we have designed novel anti-gene ONs (AGOs), directly targeting the *HTT* gene through binding of the trinucleotide-repeat region in genomic DNA, and showed efficient downregulation of mRNA and protein in HD patient fibroblasts [20]. The ONs, 12 to 19 nucleotides in length, with phosphorothioate (PS) backbone, were designed as mixmers of DNA and Locked Nucleic Acid (LNA), thereby improving the ON's capacity of strand invasion into double-strand DNA [21] and subsequent inhibition of transcription.

Although AGOs decreased *HTT* mRNA and protein levels in patient-derived fibroblasts, neither delivery of the ONs nor their downregulating effect in human neural stem cells (NSCs) or neurons was investigated. Until recently, research with this aim in focus was hampered by the inaccessibility of the viable neurons from patients. Fortunately, the discovery of induced pluripotent stem cells (iPSCs) [22–24], together with the development of neural differentiation protocols [25], made it possible to address this issue. This approach has been used to generate *in vitro* models for several genetic as well as sporadic conditions [26–31]. In particular, a number of studies have reported that iPSCs reprogrammed from HD patient samples and subsequently differentiated to either striatal or cortical neurons can be used for disease modeling and for evaluating downstream effects of gene correcting approaches [32–35].

In this report, we neuralized HD patient-derived iPSCs to investigate the transcriptional inhibition potency of CAG19, a 19-nucleotide DNA/LNA mixmer ON with PS backbone, during neural *in vitro* development. We demonstrate efficient downregulation of *HTT* mRNA following both magnetofection as well as naked delivery, later termed gymnosis, without adverse effects on the capacity of NSCs to organize into neural rosette structures or on the maintenance of the NSC pool. Furthermore, the *HTT* mRNA and protein inhibition potency of CAG19 is confirmed in neurons without off-target effects on four additional CAG•CTG trinucleotide repeat-containing genes, *POU3F2, ATN1, ATXN2, AR,* and two CUG repeat-containing transcripts, *BRI3BP* and *DMPK*. These results further validate LNA/DNA PS AGOs as a potent therapeutic strategy for transcriptional inhibition of the *HTT* gene.

## Methods

### Oligonucleotides

DNA/LNA ONs were synthesized at the Nucleic Acid Center, University of Southern Denmark, as previously reported [20]. The CAG19 ON has a PS backbone and was designed to target the CTG repeats in the DNA template strand of the *HTT* gene. The sequence of CAG19 is 5′-cAgCAgCAgCAgCAgCAgc with LNA bases written in capital letters while DNA in small. Two DNA/LNA mixmers with PS backbone nontargeting scrambled repeat ONs were synthesized and used as controls: SCR14 (5′-gAcGAcGAcGAcGA) and SCR19 (5′-gAcGAcGAcGAcGAcGAcg). All cells used in this report are commercially available and therefore no special permits are needed. The report is not a clinical trial.

### Culture of iPSC lines

The following cell lines were obtained from the National Institute of Neurological Disorders and Stroke (NINDS) Human Cell and Data Repository at the Coriell Institute for Medical Research and the NINDS Human Cell and Data Repository at RUCDR Infinite Biologics: ND41658 and ND42223. ND41658 (WT iPSC line) harbors 17/18 CAG•CTG repeats and ND42223 (HD iPSC line) harbors 109 CAG•CTG repeats. Cells were cultured under feeder-free conditions in mTeSR™1 culturing systems (STEMCELL™ Technologies) at 37°C in a humidified atmosphere of 5% CO_2_ in air. Mycoplasma test was performed using previously described protocol [36] as well as during confocal imaging. The detection revealed no mycoplasma contamination.

### Neural induction of iPSC lines

Neural induction was initiated with the formation of embryoid bodies (EBs) using AggreWell™ 800 plates (STEMCELL Technologies) and previously described neural maintenance media [25] (NMM) supplemented with 10 μM SB43154 (STEMCELL Technologies) and 10 μM Dorsomorphin (STEMCELL Technologies). After 6–8 days, EBs were collected, replated on human recombinant laminin 521 (BioLamina)-coated plates and maintained in NMM until the appearance of neural rosette structures. NSCs were then expanded by supplementing NMM with 20 ng mL^−1^ fibroblast growth factor 2 (FGF2) (PeproTech), which was withdrawn after 4 days. Cultures were manually picked or passaged using Dispase (STEMCELL Technologies) and maintained in NMM until frozen at day 23–30 postinitiation of neural induction.

For neural differentiation, BrainPhys™ Neuronal Media (STEMCELL Technologies) supplemented with B27™ (Gibco), 2 mM GlutaMAX™ (Gibco), 50 U mL^−1^ Pen/Strep, 200 nM ascorbic acid (PeproTech), 20 ng mL^−1^ human recombinant brain-derived neurotrophic factor (BDNF) (STEMCELL Technologies), and 20 ng mL^−1^ human recombinant glial-derived neurotrophic factor (GDNF) (STEMCELL Technologies) were used.

### Gymnotic delivery of ON

ONs (final concentration of 2 μM) were added into the culturing media during the neural induction and maturation progress. At indicated time points, cells were collected using Accutase (STEMCELL Technologies) and stored in 350 μL RNA Protect (Qiagen).

### ON magnetofection in NSCs

NSC and early neurons were transfected using NeuroMag Transfection reagent (OZ Biosciences) according to the manufacturer's protocol. Around 5 × 10^4^ cells were cultured in NMM and transfected 24 h postseeding with ON formulated with three different volume ratios of transfection reagent. The final concentration of the ONs was 100 nM. Forty-eight hours posttransfection, 350 μL RNA Protect (Qiagen) was added to the cells for storage.

### Allele-specific PCR of gDNA

gDNA was isolated using DNeasy (Qiagen) according to the manufacturer's protocol. The Hot-StarTaq Master Mix Kit (Qiagen) was used for the PCR according to the manufacturer's protocol. Detailed information can be found in the Supplementary Table S1.

Gel electrophoresis was performed using 1% Agarose gel in 1 × Tris-Acetate-EDTA (TAE) buffer (40 mM Tris, 20 mM acetate, and 1 mM EDTA, pH 8.3), at 90 V for 1 h.

### Quantitative reverse transcriptase PCR

Total RNA was isolated from iPSC, NSCs, and neurons using Qiagen RNeasy (Qiagen) according to the manufacturer's protocol.

For analysis of neural induction, cDNA was synthesized from 1 μg of total RNA using the RevertAid H Minus First-Strand cDNA Synthesis Kit (Thermo Fisher Scientific). *OCT4* and *PAX6* mRNA levels were analyzed using Universal SYBR Green Supermix (Bio-Rad) according to the manufacturer's instructions on the StepOnePlus^®^ Real-time PCR system (Applied Biosystems, Sweden). The relative level of gene expression was determined with d0 as the calibrator and *RPLP* and *GUSB* as endogenous references.

Analysis of the *HTT, ATN1, POU3F2*, *ATXN2, AR, BRI3BP,* and *DMPK* mRNA levels was performed using the QuantiFast^®^ Multiplex RT-PCR Kit (Qiagen) according to the manufacturer's instructions using 10 or 20 ng of total RNA. Annealing temperature for *HTT, ATN1, POU3F2, ATXN2, AR,* and *BRI3BP* was set to 60°C and for *DMPK* it was set to 55°C. StepOnePlus Real-time PCR system (Applied Biosystems, Sweden) was used for *HTT* and CFX96 Touch Real-Time PCR Detection System (Bio-Rad Laboratories) for *ATN1, POU3F2, ATXN2, AR, BRI3BP,* and *DMPK*. The relative level of gene expression was determined using the ΔΔCt method, with nontreated as the calibrator and *HPRT1* as endogenous reference.

All the primer pairs and TaqMan probes can be found in Supplementary Table S1.

### Immunofluorescence analysis

iPSCs were plated onto Matrigel (Corning)-coated coverslips and cultured until confluency in mTeSR1™ culturing system (STEMCELL Technologies). NSCs/neurons were plated on human recombinant laminin 521 (BioLamina)-coated coverslips and maintained in NMM or supplemented BrainPhys Neuronal Media (STEMCELL Technologies) until day 22 or day 38–53 postinduction, respectively. The cells were fixed with 4% paraformaldehyde/phosphate-buffered saline (PBS) for 20 min, washed in 0.1% Tween 20/PBS, and permeabilized with 0.25% Triton X-100/PBS for 10 min and blocked by 10% FBS/0.1% Tween 20/PBS for 1 h. Primary and secondary antibodies (Abs) (Supplementary Table S2) diluted in blocking solution were added for incubation overnight and 1 h, respectively, each followed by washes in 0.1% Tween 20/PBS. Nuclei were counterstained with DAPI (1:10,000, Thermo Fisher Scientific). Slides were mounted, and confocal imaging was performed on LSM META 710 Scanning Confocal (Zeiss). The raw images were exported as TIFF files by using Zen Lite software (Zeiss Microscopy). ZEN Lite software was used to measure the diameter of rosette structures. The number of Ki67-, PAX6-, and S100-positive cells were counted either using Fiji software [37] or manually.

### Western blotting

Cells were lysed and total protein extracted using the Minute™ Total Protein Extraction Kit for Animal Cultured Cells and Tissues (Invent Biotechnologies, Inc.) according to the manufacturer's protocol. The Pierce BCA Protein Assay Kit (Thermo Fisher Scientific) was used to determine protein concentration.

Proteins were separated on NuPAGE™ 3%–8% Tris-Acetate Gels (Invitrogen) (for detection of HTT and GAPDH) or NuPAGE 4%–12% Bis-Tris Gel (Invitrogen) (for detection of cleaved caspase 3) at 150V for 1 h and electrotransferred onto polyvinylidene difluoride (PVDF) membranes (iBlot^®^ Gel Transfer Stacks PVDF, Invitrogen) using iBlot system (Invitrogen). The membranes were blocked using 5% nonfat dry milk diluted in 0.1% Tween 20/PBS. Primary and secondary Abs (Supplementary Table S3) diluted in blocking solution were added for incubation overnight at 4°C and 1 h RT, respectively. Primary Ab directed against HTT detects both mHTT (upper band) and wtHTT (lower band) protein. The bands were detected using SuperSignal™ West PicoPLUS Chemiluminescent Substrate (Thermo Scientific) and visualized using ImageQuant™ LAS4000 (GE Health care). Each technical treatment replicate was blotted three times and differences in HTT and GAPDH protein levels were quantified using ImageJ software.

### Statistical analysis

**FIG. 1.**
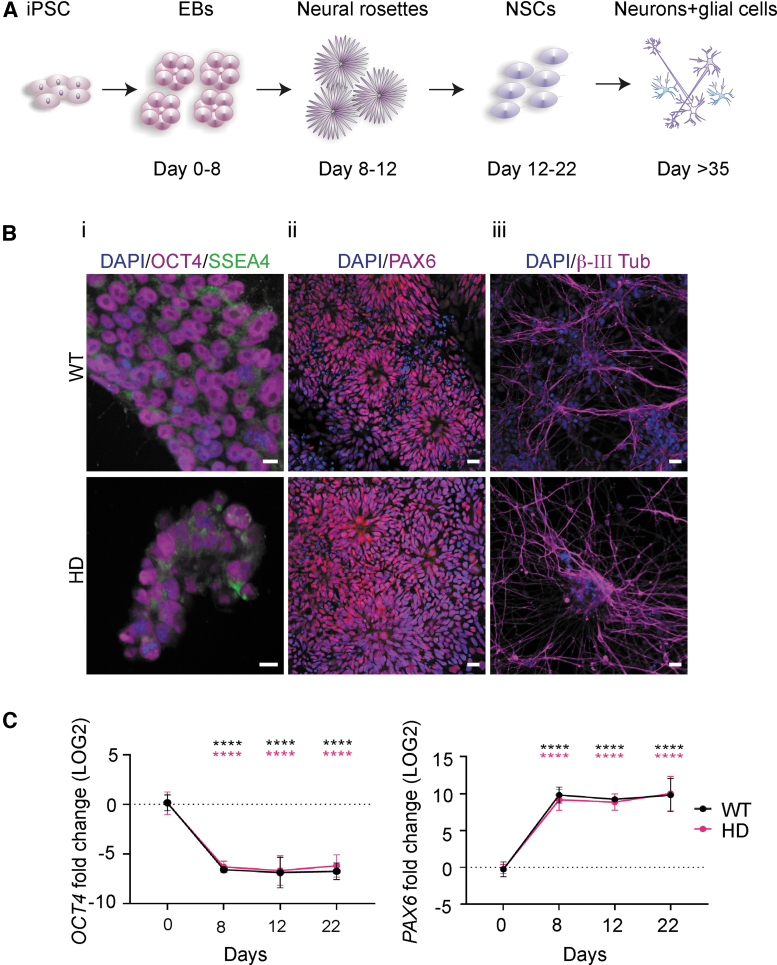
WT and HD patient-specific iPSCs readily differentiate into NSCs and subsequently neurons. **(A)** Schematic illustration of the main steps of the used method for generating NSCs from iPSC cultured as EBs. **(B)** (**i**) Representative immunocytochemistry images of WT and HD iPSC lines at d0 confirming presence of pluripotency markers OCT4 (*violet*) and SSEA4 (*green*). Nuclei were counterstained with DAPI (*blue*). Scale bars represent 10 μm. (**ii**) Neural rosette structures become prominent during the progress of neural differentiation of iPSCs and are positive for PAX6 (*violet*). Nuclei were counterstained with DAPI (*blue*). Scale bars represent 50 μm. (**iii**) Further differentiation results in the appearance of neuron-specific β-III tubulin-positive neurons (*violet*). Nuclei were counterstained with DAPI. Scale bars represent 20 μm. **(C)** During the process of differentiation both WT and HD iPSC lines terminate the pluripotency program and initiate neural differentiation. The *OCT4* and *PAX6* mRNA levels in both WT (*black*) and HD (*magenta*) lines were analyzed using RT-qPCR during the neural induction progress, that is, at day 0, 8, 12, and 22. Data are represented as mean and SD. Statistical analysis was performed using two-way ANOVA (****P < 0.0001). NSC, neural stem cell; EB, embryoid body; iPSC, induced pluripotent stem cell; WT, wild-type.

**FIG. 2.**
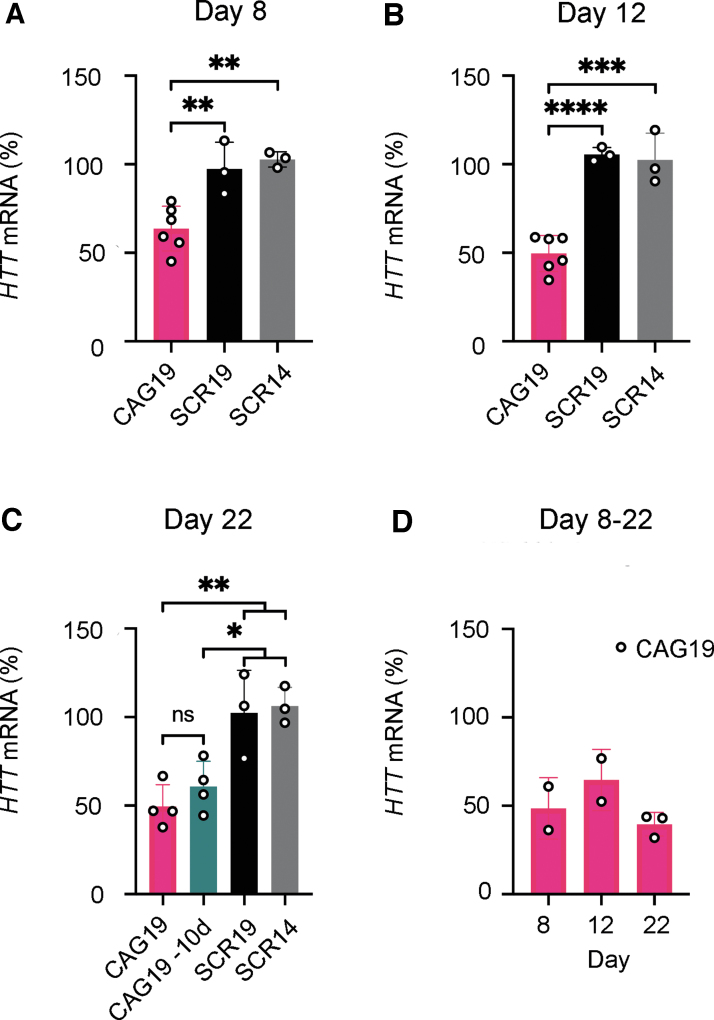
Gymnotic delivery of CAG19 induces efficient downregulation of *HTT* gene expression during the neural induction process. SCR14, SCR19, and CAG19 ONs (2 μM) were delivered using gymnosis into HD and WT lines during the progress of neural induction. The *HTT* mRNA expression, in both HD **(A–C)** and WT **(D)** cell lines, was analyzed at day 8, 12, and 22. The sample CAG19–10d represents the long-term effects of CAG19 on the *HTT* mRNA levels. The expression of *HTT* mRNA was normalized to *HPRT1* and the expression in nontreated cells was set to 100. Data are represented as mean and SD, and the symbols indicate the number of separate experiments. Statistical analysis was performed using one-way ANOVA Tukey's multiple comparisons test (*P < 0.05, **P < 0.01, ***P < 0.001, ****P < 0.0001 and ns indicates nonsignificant differences).

**FIG. 3.**
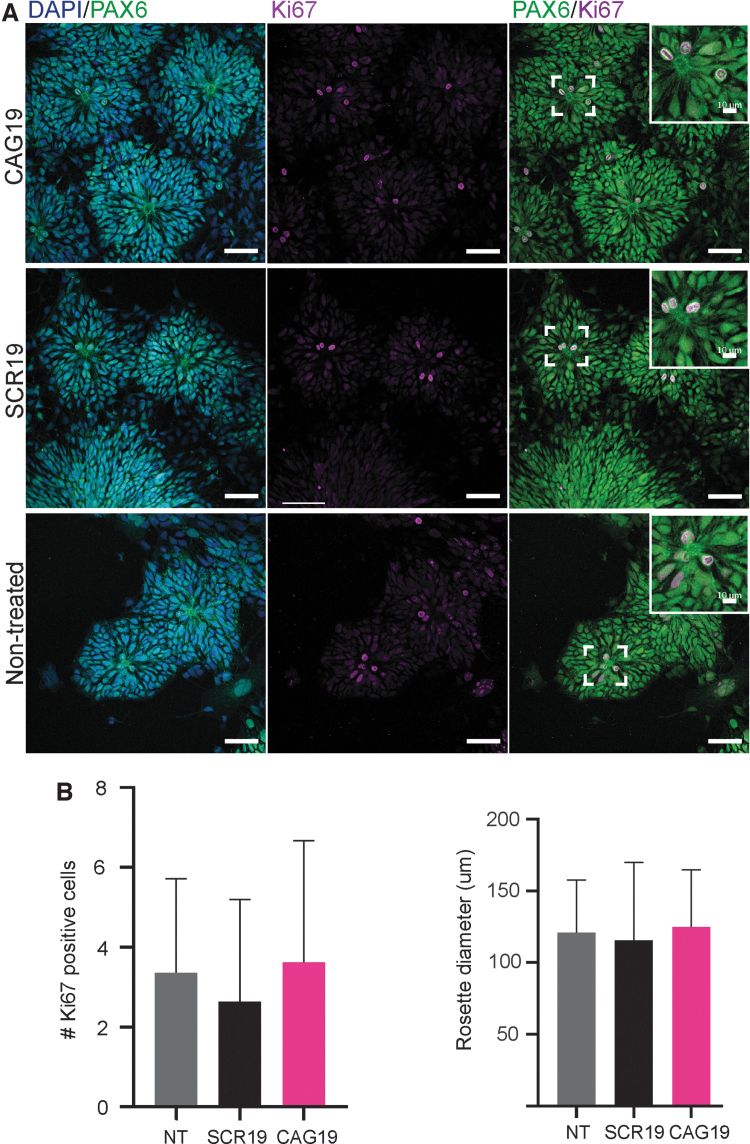
CAG19 does not have adverse effect on *in vitro* NSC organization into neural rosette structures and NSC self-renewal. **(A)** Representative images of HD line confirming presence of neural rosette structures and self-renewal of NSCs in nontreated SCR19 and CAG19-treated cells. NSCs stain positive for PAX6 (*green*) and the cell cycle marker Ki67 (*violet*). Nuclei were counterstained with DAPI (*blue*). Scale bars represent 20 μm and scale bars in *insets* represent 10 μm. **(B)** Quantification of the size of neural rosette structures and the number of PAX6/Ki67-positive cells/neural rosette structure in nontreated, SCR19 and CAG19-treated cells. The diameter and the number of Ki67-positive cells was quantified in 35 neural rosette structures per experiment (*n* = 2). Data are represented as mean and SD.

Statistical analysis was conducted using GraphPad Prism 8.4.3. Two-way ANOVA (Fig. 1) was used to assess differences in *OCT4* and *PAX6* levels during neural induction progress and between WT and HD genotype (****P < 0.0001, ns indicates nonsignificant). One-way ANOVA (Figs. 2 and 3), Tukey's multiple comparison test, assessed significant differences among SCR14-, SCR19-, and CAG19-treated groups (*P < 0.05, **P < 0.01***P < 0.001, ****P < 0.0001 and ns indicates nonsignificant differences).

**FIG. 4.**
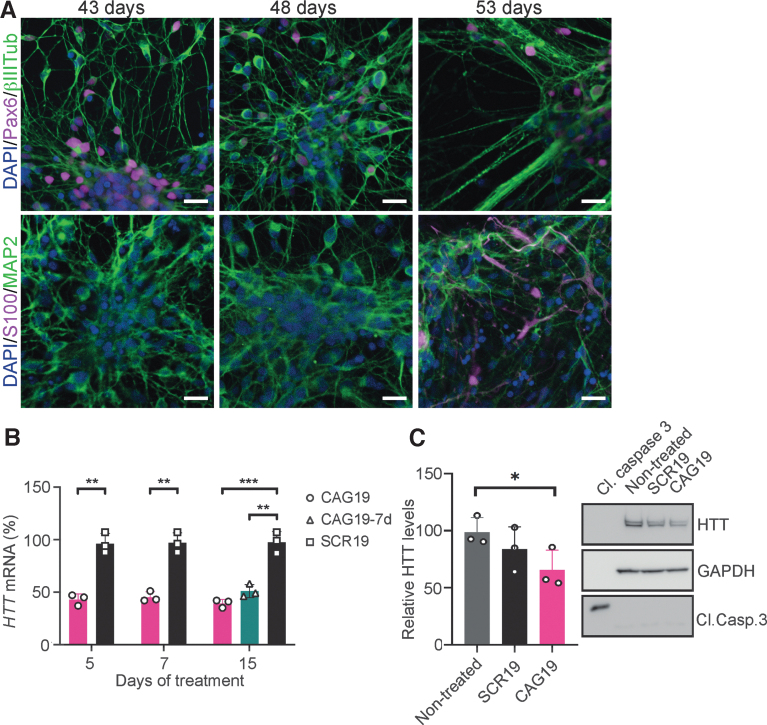
Gymnotic delivery of CAG19 induces efficient downregulation of *HTT* gene expression during the differentiation progress. **(A)** Representative immunocytochemistry images (*n* = 2) of maturing neural population at day 43, 48, and 53 postneural induction confirming presence of PAX6 (*violet*, *upper panel*), neuron-specific β-III tubulin- (*green*, *upper panel*), MAP2ab-positive neurons (*green*, *lower panel*), and S100-positive astrocytes (*violet*, *lower panel*). Nuclei were counterstained with DAPI (*blue*). Scale bars represent 20 μm. **(B, C)** SCR19 and CAG19 ONs (2 μM) were delivered into HD line during the progress of differentiation. **(B)** *HTT* mRNA levels in HD cell line were analyzed at day 43 (5 days of treatment), 45 (7 days of treatment), and 53 (15 days of treatment) of neural induction. The sample CAG19–7d represents the long-term effects of CAG19 on the *HTT* mRNA levels. The analysis of *HTT* mRNA was performed using primer probe sets spanning regions downstream of CAG•CTG repeats, normalized to *HPRT1*, and the expression in nontreated cells was set to 100%. Data are represented as mean and SD and the symbols indicate the number of separate experiments. Statistical analysis was performed using unpaired two-tailed Student's *t*-test between groups in the same day (**P < 0.01, ***P < 0.001). **(C)** Western blot analysis of HTT and cleaved Caspase 3 proteins in maturing neural cultures following CAG19 treatment. Cleaved caspase 3 (cl. Casp. 3) was used to investigate whether the CAG19 itself or its effect on HTT resulted in apoptosis. The relative HTT protein levels quantified using ImageJ and normalized to GAPDH are shown. Data are represented as mean and SD and the symbols indicate the number of technical replicates. Statistical analysis was performed using Student's *t*-test (*P < 0.05).

**FIG. 5.**
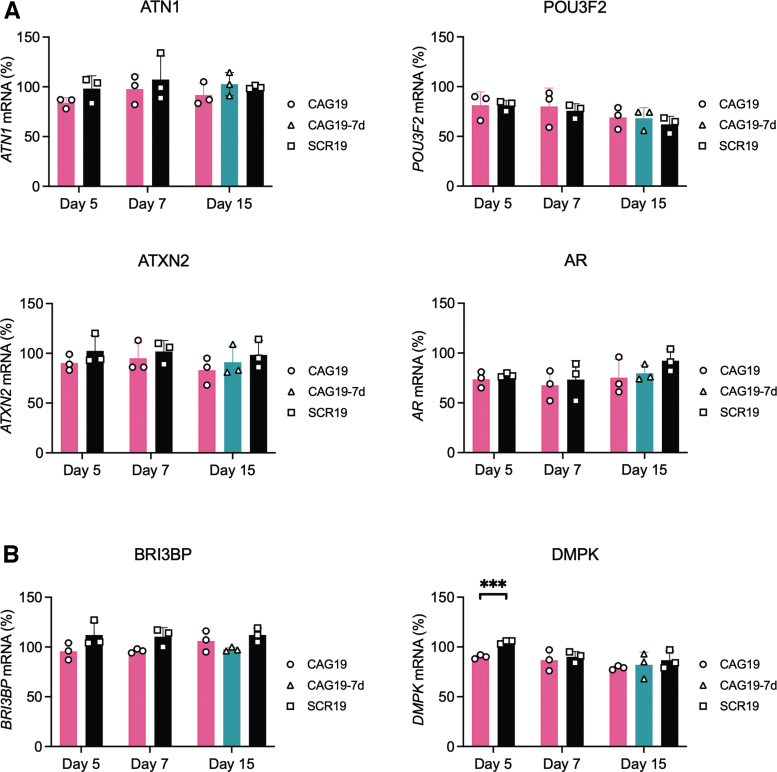
CAG19 ON does not affect **(A)** *ATN1*, *POU3F2*, *ATXN2, AR* or **(B)** *BRI3BP* and *DMPK* gene expression during the differentiation progress. SCR19 and CAG19 ONs (2 μM) were delivered into HD line during the progress of differentiation. mRNA expression was analyzed at day 43 (5 days of treatment), 45 (7 days of treatment), and 53 (15 days of treatment) of neural induction (*n* = 3). The sample CAG19–7d represents the long-term effects of CAG19 on the *HTT* mRNA levels. The analysis of mRNA levels was performed using primer probe sets spanning regions downstream of CAG•CTG repeats, the results were normalized to *HPRT1*, and the expression in nontreated cells was set to 100. Data are represented as mean and SD and the symbols indicate the number of separate experiments. Statistical analysis was performed using unpaired two-tailed Student's *t*-test between groups in the same day (***P < 0.001).

The differences in *HTT* (Fig. 4B*), ATN, POU3F2, ATXN2, AR, BRI3BP,* and *DMPK* mRNA levels at d43–d53 (Fig. 5) and HTT protein levels between SCR19 and CAG19 (Fig. 4C) were assessed using unpaired Student's *t*-test.

The data are represented as mean and SD and the symbols indicate either the number of separate experiments (Fig. 2) or neural differentiations (Figs. 4 and 5).

## Results

### Healthy control and HD patient-derived iPSCs are differentiated into neural lineage

Integration-free WT and HD iPSC lines were exposed to a stepwise differentiation protocol mimicking human neural development (Fig. 1A) to be used as a model for investigating the efficiency of the CAG19 ON to downregulate *HTT* gene expression in the early stages of neural development. Before differentiation, the pluripotency of iPSC lines was confirmed using immunofluorescence and confocal microscopy. Both WT and HD iPSC lines were positive for SSEA4, a glycolipid carbohydrate antigen expressed on the surface of human pluripotent cells, and the cell lines exhibited intense nuclear immunostaining for pluripotency marker OCT4 (Fig. 1B: panel i, and Supplementary Fig. S1A). The WT and HD iPSC lines were cultured as EBs and neuralized by dual inhibition of SMAD combined with retinoid signaling to differentiate the iPSC lines into the neuroectodermal fate. At day 8, 12, and 22 postneural induction, confirmation of directed iPSC differentiation was investigated by expression levels of *OCT4* and *PAX6*. As expected, already on day 8, both lines showed *PAX6* neuroectodermal acquisition while *OCT4* expression was decreased (Fig. 1C), suggesting that the cells have terminated the pluripotency program and initiated neural differentiation. Furthermore, on day 8, both lines formed neural rosette structures with a morphology characteristic of early neuroepithelium and a feature of iPSC-derived NSCs [38–41]. This was further confirmed by immunofluorescence analysis showing the presence of PAX6 (Fig. 1B: panel ii and Supplementary Fig. S1B). Subsequently, NSCs differentiated into neurons, as confirmed by the presence of early neuron-specific β-III tubulin-positive cells (Fig. 1B: panel iii).

Taken together, these results demonstrate the successful differentiation of both WT and HD iPSC lines into NSCs and, subsequently, neurons.

### Gymnoticaly delivered CAG19 ON downregulates *HTT* mRNA during the iPSC differentiation

Having confirmed the differentiation potency of WT and HD iPSC lines, we investigated whether the CAG19 ON, designed to target the template strand of *HTT* repeat region, efficiently downregulates *HTT* gene expression during the neural induction process. This ON has previously shown to decrease the *HTT* mRNA levels with some selectivity toward mutant allele in comparison to other investigated shorter ONs [20].

The CAG•CTG repeat-expanded alleles are unstable in both the germline and somatic cells [42–44], which can result in the expansion, deletion, and contraction of the repeat region [43]. Therefore, we confirmed the presence of an expanded mt allele in the HD iPSC line, and in the first stages of neural induction (Supplementary Fig. S2).

Since ON delivery to the central nervous system (CNS) typically involves direct injection/infusion techniques, such as intrathecal or intracerebroventricular infusion [45], the most clinically relevant uptake mechanism to study *in vitro* would be the gymnosis delivery strategy [46]. Therefore, WT and HD iPSC lines were treated with CAG19 ON, using gymnosis. Control ONs having the same chemical modification, but nontargeting sequences (scrambled repeat ONs, SCR14, and SCR19) were also delivered using gymnosis. SCR14 was previously tested on HD fibroblasts together with another scrambled ON and several mismatch controls, all showing no significant effect on *HTT* mRNA and HTT protein [20], whereas SCR19 was included in this study to have a control with the same number of nucleotides as CAG19. Cell treatment started at day 0 of neural induction and maintained during the progress of differentiation until day 22 (Fig. 1A for developmental stages). The total concentration of ONs was kept at 2 μM by the addition of ONs each time the culturing medium was exchanged. To determine the expression levels of the *HTT* gene, the treated cells were harvested at three separate time points, that is, end of neural induction of EBs (day 8), prominent neural rosette structures (day 12), and NSCs and early neurons (day 22). *HTT* mRNA levels were significantly decreased following the CAG19 ON treatment, compared with SCR14 and SCR19 at all analyzed developmental stages in the HD cell line with a maximum of 50% downregulation at day 12 and 22 (Fig. 2A–C). This indicates that the effect observed in the CAG19-treated cells results from specific targeting of the *HTT* gene. Since CAG19 cannot discriminate between the wt and mt *HTT* alleles, that is, allele-nonspecific, *HTT* mRNA levels were also decreased in the WT cell line (Fig. 2D).

To evaluate the long-term effects of the CAG19, the HD cells were treated during the first 12 days with the ON, followed by cultivation in CAG19-free media for the following 10 days. Cells were harvested for analysis of *HTT* gene expression at day 22. There is an indication that the *HTT* mRNA expression increases after omitting CAG19 from the culturing media for 10 days. However, this potential alteration, that is, the difference of *HTT* mRNA levels between the CAG19 and CAG19–10d treatments, did not reach statistical significance (Fig. 2C, CAG19 -10d).

These results indicate that the CAG19 ON efficiently downregulates *HTT* gene expression during the directed neural differentiation of iPSCs.

### CAG19 ON treatment does not affect *in vitro* NSC organization into neural rosette structures or the NSC pool

Since ONs may cause adverse effects by either affecting the target or through off-targeting, we next investigated whether the CAG19 induces *in vitro* neurodevelopmental toxicity. For this purpose, the capacity of NSCs to self-organize into neural rosette structures, generated during a critical morphogenetic process during both *in vivo* and *in vitro* neural development [38–41], was assessed as functional/morphologic endpoint. As in the above-described experiments, gymnotic treatment of cells with CAG19 was initiated at day 0 and maintained during the differentiation progress. Immunofluorescence analysis, combined with confocal microscopy, revealed the presence of PAX6-positive neural rosette structures regardless of CAG19 treatment (Fig. 3A, DAPI/PAX6).

During this developmental stage, neural rosette structures serve as a niche for the maintenance and proliferation, that is, self-renewal, of NSCs with the capacity to differentiate into neurons and glia during subsequent developmental stages. To investigate whether the CAG19 affects NSC proliferation within neural rosette structures, and consequently, the future neural pool, that is, number of neuronal and glial cells, immunofluorescence analysis using the cell cycle marker Ki67 was employed. The Ki67-positive cells were observed in the apical part of neural rosette structures (Fig. 3A, PAX6/Ki67), suggesting active proliferation of NSCs. Furthermore, quantification of Ki67-positive cells localized solely to the apical part of rosette structures indicates similar number of Ki67-positive cells regardless of CAG19 treatment (Fig. 3B). In addition, the size, that is, diameter, of the rosette structures was measured suggesting that CAG19 ON does not affect NSC proliferation (Fig. 3B).

Taken together, these results suggest that the CAG19 ON does not have adverse effects on either NSC organization into neural rosette structures or NSC pool.

### CAG19 ON downregulates HTT mRNA in NSC following magnetofection

For targeted *in vitro* and *in vivo* gene therapy application, magnetofection-based technology using biocompatible nanoparticles can be used [47]. Given that magnetofection delivers genetic material to otherwise hard-to-transfect NSCs and early neurons [48–50], we sought to evaluate it as an AGO delivery strategy. Therefore, 100 nM of CAG19 ON, as well as control nontargeting SCR14 and SCR19 ONs were associated with superparamagnetic nanoparticles at different volume ratios and delivered to NSCs and neurons (d37) by application of a magnetic field. *HTT* gene expression levels were analyzed 48 h postmagnetofection revealing significantly decreased *HTT* mRNA levels (Supplementary Fig. S3).

These data indicate efficient CAG19 ON delivery using magnetofection as a delivery strategy and further confirms its *HTT* downregulating potency.

### CAG19 ON gymnotic delivery downregulates HTT mRNA in neurons without affecting the expression of other CAG•CTG repeat-containing genes

HD cells were further differentiated into neurons and astrocytes to assess the CAG19 ON potency to silence *HTT* gene expression during the neural differentiation process. BrainPhys Neuronal Medium supplemented with BDNF and GDNF was used to enhance neuronal maturation [51]. At day 43, 48, and 53 postneural induction, confirmation of neural differentiation was performed using immunofluorescence analysis combined with confocal microscopy. As expected, during all investigated developmental stages, the presence of PAX6-(45% ± 18% at day 43, 43% ± 10% at day 48, and 35% ± 12% at day 53), β−III Tub-(ratio between %area of β−III Tub and DAPI: 1.48 ± 0.29 at day 43, 1.56 ± 0.48 at day 48, and 1.4 ± 0.3 at day 53), and mature neuron-specific microtubule-associated protein 2, isoforms a and b (MAP2ab)- (ratio between %area of MAP2 and DAPI: 1.73 ± 0.19 at day 43, 1.4 ± 0.53 at day 48, and 2.2 ± 1 at day 53), and astrocyte-specific S100 calcium-binding protein (S100) (0.19% ± 0.19% at day 43, 0.22% ± 0.38% at day 48, and 0.72% ± 0.8% at day 53)-positive cells was confirmed (Fig. 4A, upper and lower panel). These results indicate the presence of NSCs and neurons but also astrocytes at indicated time points.

At these developmental stages, the culture, predominated by young and mature neurons sensitive to any microenvironmental changes, was treated using gymnosis with 2 μM CAG19 ON and nontargeting SCR19 ON, as it has the same number of nucleotides as CAG19. The treatment was initiated at day 38 of neural induction and maintained for 15 days during maturation. To determine the expression levels of the *HTT* gene, treated cells were harvested after 5, 7, and 15 days (day 43, 45, and 53 postinduction, respectively). *HTT* mRNA levels were significantly decreased following CAG19 ON, in comparison to SCR19, treatment during all analyzed time points, with a maximum of 61% downregulation at day 15 (Fig. 4B). Furthermore, the CAG19 ON long-term effect was evaluated, revealing the remaining *HTT* mRNA downregulation to be significant and stable during the assessed time (Fig. 4B, 15d, CAG19–7d).

Based on the persistent *HTT* mRNA downregulation at this developmental stage and reported HTT-lowering effect in fibroblasts [20], we investigated the hypothesis that CAG19 ON treatment decreases HTT protein levels in the above-described cultures. Western blot was performed 15 days after initiation of the treatment. Quantification using ImageJ revealed that the HTT protein decrease is nonsignificant compared with SCR19-treated cells, while the decrease is significant compared with nontreated cells (Fig. 4C). Furthermore, the absence of cleaved caspase 3 (Cl. Casp. 3), suggests that the observed HTT protein decrease is neither dependent on, nor accompanied by apoptosis (Fig. 4C).

In addition to *HTT*, several other genes contain a region of CAG•CTG trinucleotide repeats representing potential off-targets for CAG19 ON and could result in adverse effects. By selecting four genes with either proximally or distally located CAG•CTG trinucleotide repeats, potential CAG19 ON off-targets were addressed; the POU-homeodomain transcription factor BRN2, encoded by *POU3F2*, involved in neural formation and cell fate determination [52], migration [53], neurogenesis, and positioning of cortical neurons [54,55] and the transcriptional corepressor Atrophin-1, encoded by *ATN1* and mutated in dentatorubral–pallidoluysian atrophy; Ataxin 2, an RNA-binding protein encoded by *ATXN2*, and implicated in amyotrophic lateral sclerosis and spinocerebellar ataxia-2 [56]; and androgen receptor, encoded by *AR* and implicated in transcriptional regulation and proliferation [57]. Gymnotic treatment was performed as in the above-described experiments. Using quantitative reverse transcriptase PCR (RT-qPCR) quantification with different primer-probe sets spanning regions downstream (Fig. 5) or upstream (Supplementary Fig. S4) of the CAG•CTG repeats revealed that CAG19 ON, compared with SCR19, treatment does not significantly affect the expression levels of *POU3F2, ATN1, ATXN2,* or *AR* (Fig. 5A and Supplementary Fig. S4).

The CAG19 ON off-target effects on CUG repeat-containing transcripts, that is, steric block antisense efficiency, was investigated using two genes: BRI3-binding protein (BRI3BP) [58], with CTG•CAG repeats in exon 1 and DM1 protein kinase (DMPK), a nonreceptor serine/threonine protein kinase, with the CTG•CAG repeats in the 3′ untranslated region (3’UTR) [59]. RT-qPCR analysis revealed no significant downregulation of *BRI3BP* compared with SCR19 (Fig. 5B, panel *BRI3BP*). After 5 days we observed significant downregulation of *DMPK*, however, this effect disappeared at day 7 and 15 (Fig. 5B, panel DMPK).

These results indicate that CAG19 ON exerts a specific downregulating effect on *HTT* gene expression without affecting additionally investigated CAG•CTG repeat-containing genes or CUG repeat-containing transcripts following gymnotic delivery in the patient-specific NSCs, neurons, and astrocytes.

## Discussion

In this report, we neuralized WT and HD patient-derived iPSCs to investigate the transcriptional inhibition potency of CAG19 ON during neural *in vitro* development. Furthermore, the CAG19 ON off-targeting and its effect on neural rosette formation were assessed. To resemble intended neuronal target cells, we studied an HD patient-derived iPSC line carrying 109 repeats in the disease allele. Thus, although a 109-repeat allele is considerably shorter than most somatically expanded alleles [60], this was the largest number of repeats in an iPSC line that we could obtain.

CAG19 ON, a DNA/LNA mixmer with a PS backbone, is designed to target the *HTT* gene through binding of the trinucleotide-repeat DNA and was previously shown to efficiently downregulate expression of *HTT* mRNA and protein in HD patient fibroblasts [20]. The mechanism of action involves strand invasion into dsDNA, binding to the template strand and, consequently, transcriptional inhibition. The *HTT* mRNA downregulation is observed during several stages of neural differentiation (Figs. 2A–C, 4B) and is in accordance with the previously reported effect in HD patient fibroblasts [20]. However, the effect of the CAG19 on the HTT protein level was not pronounced (Fig. 4C). This might depend on the culture's heterogeneity, but further studies investigating this difference in detail are needed.

CAG19 ON also downregulates *HTT* expression in the WT cell line (Fig. 2D). This finding is expected since CAG19 ON is targeting the CAG•CTG repeat sequence in the first exon of *HTT* gene found in both wt and mt alleles and suggests that its mechanism of action is not dependent on the repeat length. However, the allele-nonspecific reduction of total *HTT* mRNA is shown to be well tolerated in several *in vitro* and *in vivo* studies and, importantly, in clinical trials [7–9,11,13], and thus is considered acceptable.

The anti-gene off-targeting analysis indicates unaffected mRNA expression of four other CAG•CTG repeat-containing genes. The repeats are located either proximally or distally to the promoter: *ATXN2* (<30 CAG•CTG repeats exon 1), *AR* (8–37 CAG•CTG repeats exon 1), *POU3F2* (6 CAG•CTG repeats in exon 1), and *ATN1* (15–35 CAG•CTG repeats in exon 5) (Fig. 5A). These results further confirm that the CAG19 ON´s mechanism of action is not dependent on the distance between different regulatory elements and the CAG19 ON targeted site and suggest *HTT* specificity. The CAG19 downregulating potency might depend on chromatin changes affecting the accessibility of ONs within the repeat region. Furthermore, we evaluated antisense off-target effect by selecting *BRI3BP* (9–10 repeats CTG•CAG repeats in exon 1) and *DMPK* (5–38 CTG•CAG repeats in 3'UTR). The CAG19 ON is an LNA/DNA mixmer and would therefore work as a steric block ASO. These generally mask splicing signals, AUG start codon, or polyadenylations signal to exert their function [61–63]. We observed only minor changes in the expression of *DMPK* at day 5 and no effect on *BRI3BP* (Fig. 5B). Although we did not detect any anti-gene or antisense off-targeting in this study, further study using RNA sequencing would be necessary to enable more detailed evaluation.

The CAG19 ON effect on *HTT* downregulation is persistent during the treatment period, although a nonsignificant increase in *HTT* expression is observed when investigating long-term effects in NSCs and in neurons (Figs. 2C, 4B). This is in accordance with previous results in the HD fibroblasts [20], since constant *HTT* downregulation could still be detected here for 10 and 7 days after dosing, respectively. This indicates that the ON remains active for a more extended period, which is not surprising since PS-modified ONs resist degradation by endo- and exonucleases [64,65]. In *in vivo* experiments or in clinical settings, it is reasonable to assume based on ASO treatment strategies in clinical trials, that the CAG19 ON effect will become transient as time progresses. This is advantageous if an unwanted outcome, such as excessive downregulation of either wt*HTT* or off-targets occurs.

Moreover, we investigated the CAG19 ON adverse effects related to the NSC organization into neural rosette structures, a critical morphogenetic process during neural development. The CAG19 treatment achieving 35%–50% *HTT* downregulation (Fig. 2A–C), neither compromises the emergence of neural rosette structures (Fig. 3A) nor the NSC proliferation (Fig. 3), essential for the neuronal and glial pool. These results indicate that the CAG19 ON is not adversely affecting mechanisms important for this developmental stage and could also suggest possible CAG19 ON downregulating effects on adult NSC pool, as it is proposed that a neurogenic ventricular zone persists in the adult mammalian brain [66]. However, further studies using transcriptome sequencing are needed to investigate these crucial questions in detail.

To effectively treat HD, ONs, or any other therapeutic molecules must be efficiently delivered to the cell type affected by the disorder. Stem cells, in general, and neurons, in particular, are cell types that are hard to transfect. In our experiments, CAG19 ON shows the ability to exert downregulation of *HTT* gene expression in NSCs and neurons without the aid of potentially toxic transfection reagents. Gymnotic delivery is the most clinically relevant since, in general, ONs delivered to CNS using such routes have shown half-lives of several weeks and a broad distribution across brain regions [13]. Furthermore, it shows good correlation between *in vitro* and *in vivo* results [46]. DNA targeting ONs, like other ONs, are entering cells through endocytosis [67]. Thus, the concentration of ONs is substantially reduced as it is eventually degraded. Our treatment and differentiation experiments are executed in a 3D environment during the first 8 days, that is, as EBs (Fig. 1A), but significant *HTT* mRNA downregulation is achieved (Fig. 2A). Hence, the CAG19 ON and the HD model system could also be used to investigate whether ONs are delivered to cells in the intrasphere 3D environment through exosomes, which could be important for several research fields.

In addition to gymnosis, we sought to evaluate a magnetofection-based technology [47] as an ON delivery strategy. Magnetofection can potentiate the efficacy of any vector up to several hundred-folds and allow reduction of the duration of gene delivery [47], and, in comparison to gymnosis, decreased amounts of administered ON are needed. Importantly, it can deliver genetic material to otherwise hard-to-transfect NSCs and early neurons. The achieved 40%–45% *HTT* mRNA downregulation (Supplementary Fig. S3) is consistent with the gymnosis experiments, further strengthening the anti-gene concept.

In summary, we differentiated HD patient-derived and healthy control iPSCs to investigate the transcriptional inhibition potency of AGOs during *in vitro* human neurodevelopment. CAG19 ON targets the DNA template strand of the CAG•CTG trinucleotide-repeats in the *HTT* gene. We demonstrate efficient downregulation of *HTT* mRNA following gymnosis into NSCs and neurons, suggesting that NSCs and, importantly, neurons can be targeted under conditions resembling the clinical method of choice for treating CNS disorders [45,68]. Furthermore, we show that the CAG19 ON treatment does not compromise the emergence of neural rosette structures, self-renewal of NSCs, or *ATN1*, *POU3F2, ATXN2, AR, BRI3BP,* and *DMPK* expression. Thus, these results further validate the LNA/DNA CAG19 ON-based anti-gene strategy as a potent therapeutic concept for transcriptional inhibition of the *HTT* gene.

## Supplementary Material

Supplemental data

Supplemental data

Supplemental data

Supplemental data

Supplemental data

Supplemental data
