# Supplementary material for: Oligonucleotides Targeting DNA Repeats Downregulate Huntingtin Gene Expression in Huntington's Patient-Derived Neural Model System
Source: Nucleic Acid Ther. 2021 Dec 10;31(6):443–56. doi: 10.1089/nat.2021.0021 (PMC8713517; doi:10.1089/nat.2021.0021)
Supplement: Supplemental data [file Supp_TableS1-S2.pdf]

Target mRNA	Forward primer (5'-3')	Reverse primer (5'-3')	TaqMan probe (5'-3')
OCT4	cctggagaaagagtttgagag	ggttgtggaattggttggtag	
PAX6	ggttctatttgggaaggtatt	ctggttcggtttctcttt	
RPLP	cgacaatggcagcatctacaac	cggacaccctccaggaag	
GUSB	aatcactatgcgcatcaacaaca	ttgggatacttggaggtgtca	
HTT	gactcgaacaagcaagag	gcctttaacaaaaccttaatttc	[JOE]gaagaatcagtccaggagacc[BHQ1]
ATN1	gagcagagaagtttctgta<br>gaggactactacagtcac	ccactcctcattgacatc<br>ctccaaggtccaatgta	[HEX]tgtcgtgtcttcattcttcaggct[BHQ1]<br>[HEX]aagccactgtagaacctgcga[BHQ1]
POU3F2	gagagaggagacagaaaga<br>cctcaaatgccccaa	aggctgtagtggttagac<br>ggtcatccttttctctttc	[HEX]ctgcggtcgccatgactctc[BHQ1]<br>[HEX]aactctcaccacctccttctcc[BHQ1]
HPRT1	gagctattgtaatgaccagtc	tgaccaaggaaagcaaag	[6FAM]tgccagtgtcaattatatcttccacaa[BHQ1]
AR	aatcccacatcctgctcaag	aagtccacgctcaccatg	[HEX]actccgtgcagcctattgcga[BHQ1]
ATXN2	tcgccacagaatagtttccc	ggcagttggatgagaaggaa	[HEX]cggctgaacgtgagaaggatgga[BHQ1]
BRI3BP	ctgggagtggatatgttcgtg	ctgggctgaaatactgggac	[6-FAM]actgtggaaagtctggaccgagc[BHQ1]
DMPK	gcctgcttactcgggaaa	ggtcctgtagcctgtcag	[JOE]cctcctcacttgcgctgctctcgg[BHQ1]
HPRT1 BRI3BP	tgctgaggatttggaaaggg	acagagggctacaatgtgatg	[HEX]aggactgaa/ZEN/cgtcttgctcgagatg[IABkFQ]
HPRT1 DMPK	gcctaagatgagagttc	cacagaactagaacattgata	[6-FAM]atctggagtcctattgacatcgcc[BHQ1]

Target gene	Forward primer (5'-3')	Reverse primer (5'-3')
HTT	gcgaccctggaaaagctgatgaag	cggctgaggaagctgaggag

Supplementary Table 1. List of primers and TaqMan probes used for RT-qPCR and allele specific PCR amplifying over CAG•CTG repeats in *HTT* gene. JOE, HEX, and 6-FAM are three fluorophores having different emission spectra and with Black Hole Quencher (BHQ1) and ZEN^{TM} with Iowa Black® FQ (IABkFQ) used as the quenchers.

Antigen	Clone (symbol)	Catalogue number	Dilution	Supplier
**OCT-3/4**	H-134	sc-9081	1:200	Santa Cruz
**SSEA4**	MC813	ab16287	1:50	Abcam
**PAX6**		ab2237	1:200	Millipore
**MAP2ab**	MT-08	SM3121P	1:200	Acris GmbH
**S100**		Z0311	1:300	Dako
**Ki67**		sc-23900	1:200	Santa Cruz
**β-Tubulin III**	Tuj-1	MAB1195	1:500	R&D systems
**Alexa Flour®488 IgG (H+L) Donkey anti mouse**		A21202	1:500	Life Technologies
**Alexa Flour®568 Donkey anti-rabbit**		A10042	1:500	Life Technologies

Supplementary Table 2. List of primary and secondary antibodies used for immunofluorescence analysis.
