# Supplementary material for: Oligonucleotides Targeting DNA Repeats Downregulate Huntingtin Gene Expression in Huntington's Patient-Derived Neural Model System
Source: Nucleic Acid Ther. 2021 Dec 10;31(6):443–56. doi: 10.1089/nat.2021.0021 (PMC8713517; doi:10.1089/nat.2021.0021)
Supplement: Supplemental data [file Supp_TableS3.pdf]

Antigen	Catalogue number	Dilution	Supplier
**HTT**	ab109115	1:1000	Abcam
**GAPDH**	MAB374	1:1000	Millipore
**Cleaved caspase 3**	ab2302	1:1000	Abcam
**Goat anti-Mouse IgG + HRP**	32430	1:200	Invitrogen
**Donkey anti-rabbit IgG + HRP**	NA934V	1:5000	GE Healthcare
**Recombinant human cleaved Caspase 3 protein**	Ab52314		Abcam

Supplementary Table 3. List of primary and secondary antibodies and recombinant protein used for western blot analysis.
