# Supplementary material for: Oligonucleotides Targeting DNA Repeats Downregulate Huntingtin Gene Expression in Huntington's Patient-Derived Neural Model System
Source: Nucleic Acid Ther. 2021 Dec 10;31(6):443–56. doi: 10.1089/nat.2021.0021 (PMC8713517; doi:10.1089/nat.2021.0021)
Supplement: Supplemental data [file Supp_FigS1.pdf]

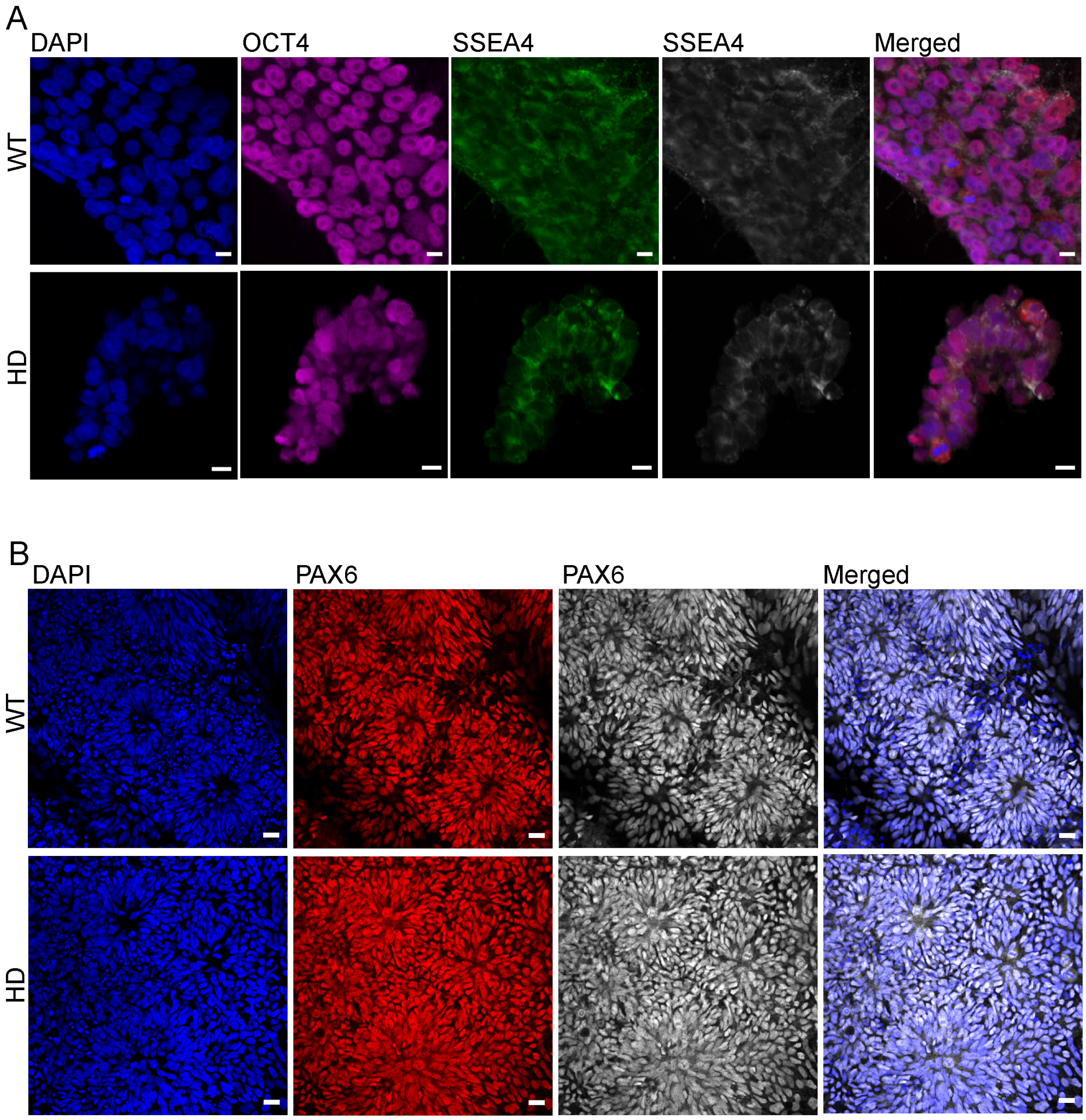

Supplementary Figure 1. WT and HD-patient specific iPSCs readily differentiate into neural stem cells. (A) Immunocytochemistry images of WT and HD iPSC lines at d0 confirming presence of pluripotency markers OCT4 (red) and SSEA4 (green, white). Nuclei were counterstained with DAPI (blue). Scale bars represent 10 µm. **(B)** Neural rosette structures are positive for PAX6 (red, white). Nuclei were counterstained with DAPI (blue). Scale bars represent 50 µm.
