# Supplementary material for: Oligonucleotides Targeting DNA Repeats Downregulate Huntingtin Gene Expression in Huntington's Patient-Derived Neural Model System
Source: Nucleic Acid Ther. 2021 Dec 10;31(6):443–56. doi: 10.1089/nat.2021.0021 (PMC8713517; doi:10.1089/nat.2021.0021)
Supplement: Supplemental data [file Supp_FigS2.pdf]

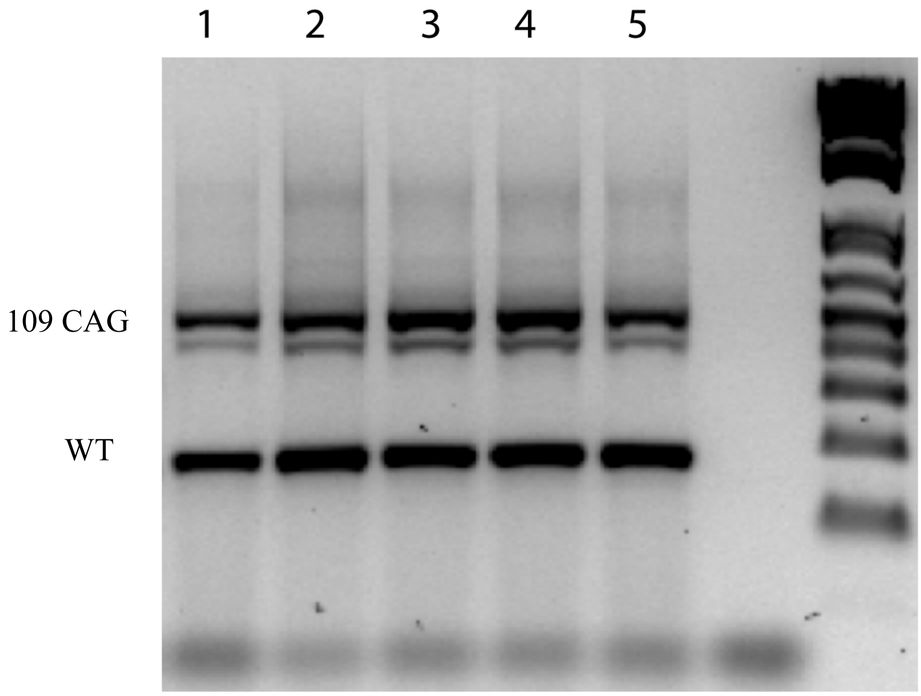

Supplementary Figure 2. Confirmation of HD line genotype. Representative agarose gel electrophoresis of allele-specific genomic DNA PCR showing repeat allele distribution of HD line during iPSC differentiation into neural stem cells. (1) HD iPSC line harbors one wt allele (wt) and one allele with the expanded repeats (109 CAG) (1) day 0; (2) at day 6 following the treatment with CAG19; (3) at day 6 non-treated; 4) at day 12 following the treatment with CAG19 (5) at day 12 non-treated (6) Negative control (water) (7) 1kb+ DNA ladder.
