# Supplementary material for: Oligonucleotides Targeting DNA Repeats Downregulate Huntingtin Gene Expression in Huntington's Patient-Derived Neural Model System
Source: Nucleic Acid Ther. 2021 Dec 10;31(6):443–56. doi: 10.1089/nat.2021.0021 (PMC8713517; doi:10.1089/nat.2021.0021)
Supplement: Supplemental data [file Supp_FigS3.pdf]

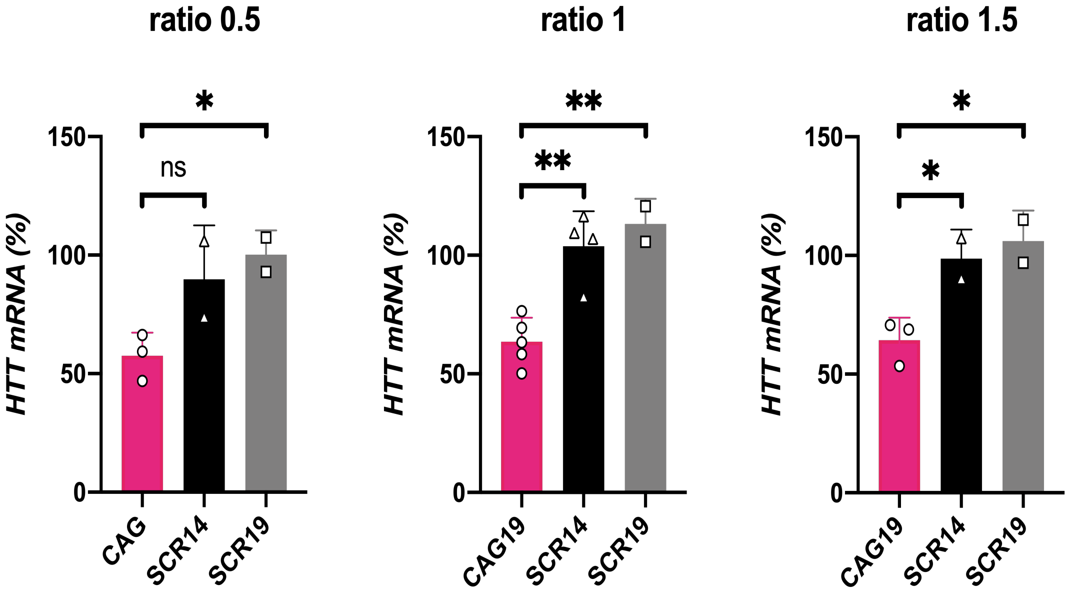

Supplementary Figure 3. Magnetofection of CAG19 induces efficient down-regulation of *HTT* gene expression. HD NSCs were transfected with SCR14, SCR19 and CAG19 using 100 nM and three different volume ratios of ON to transfection reagent (0.5, 1 and 1.5). The *HTT* mRNA expression was analyzed 48h post magnetofection. The expression of *HTT* mRNA was normalized to *HPRT1* and the expression in non-treated cells was set to 100. The data is represented as mean and SD and the symbols indicate the number of separate experiments.
