# Supplementary material for: Oligonucleotides Targeting DNA Repeats Downregulate Huntingtin Gene Expression in Huntington's Patient-Derived Neural Model System
Source: Nucleic Acid Ther. 2021 Dec 10;31(6):443–56. doi: 10.1089/nat.2021.0021 (PMC8713517; doi:10.1089/nat.2021.0021)
Supplement: Supplemental data [file Supp_FigS4.pdf]

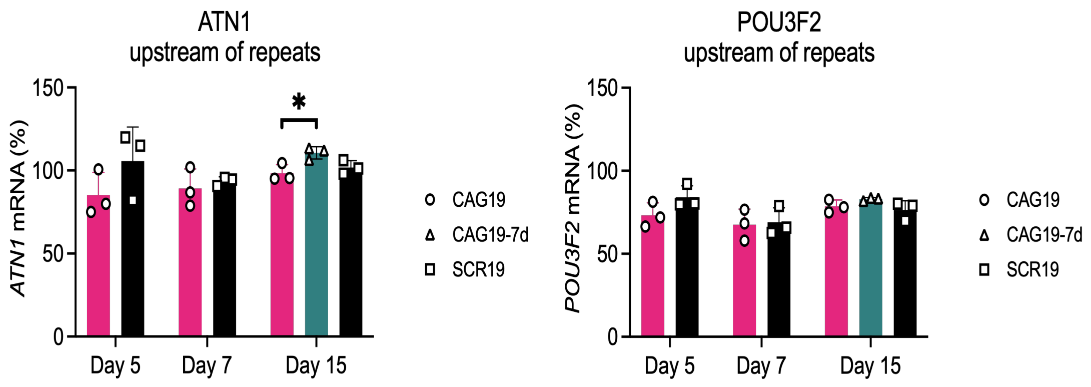

Supplementary Figure 4. CAG19 ON does not affect *ATN1* and *POU3F2* gene expression during the differentiation progress. SCR19 and CAG19 ONs (2 µM) were delivered into HD line during the progress of differentiation. *ATN1* and *POU3F2* mRNA expression was analyzed at day 43 (5 days of treatment), 45 (7 days of treatment) and 53 (15 days of treatment) of neural induction (n=3). The sample CAG19-7d represents the long-term effects of CAG19 on the *HTT* mRNA levels. The analysis of *ATN1* and *POU3F2* mRNA levels were performed using a primer probe sets spanning regions upstream of CAG•CTG repeats, were normalized to *HPRT1* and the expression in non-treated cells was set to 100. Statistical analysis was performed using unpaired Student’s t test.
